# Supplementary figures and images for: Using bioinformatics and metabolomics to identify altered granulosa cells in patients with diminished ovarian reserve
Source: PeerJ. 2020 Aug 28;8:e9812. doi: 10.7717/peerj.9812 (PMC7457930; doi:10.7717/peerj.9812)

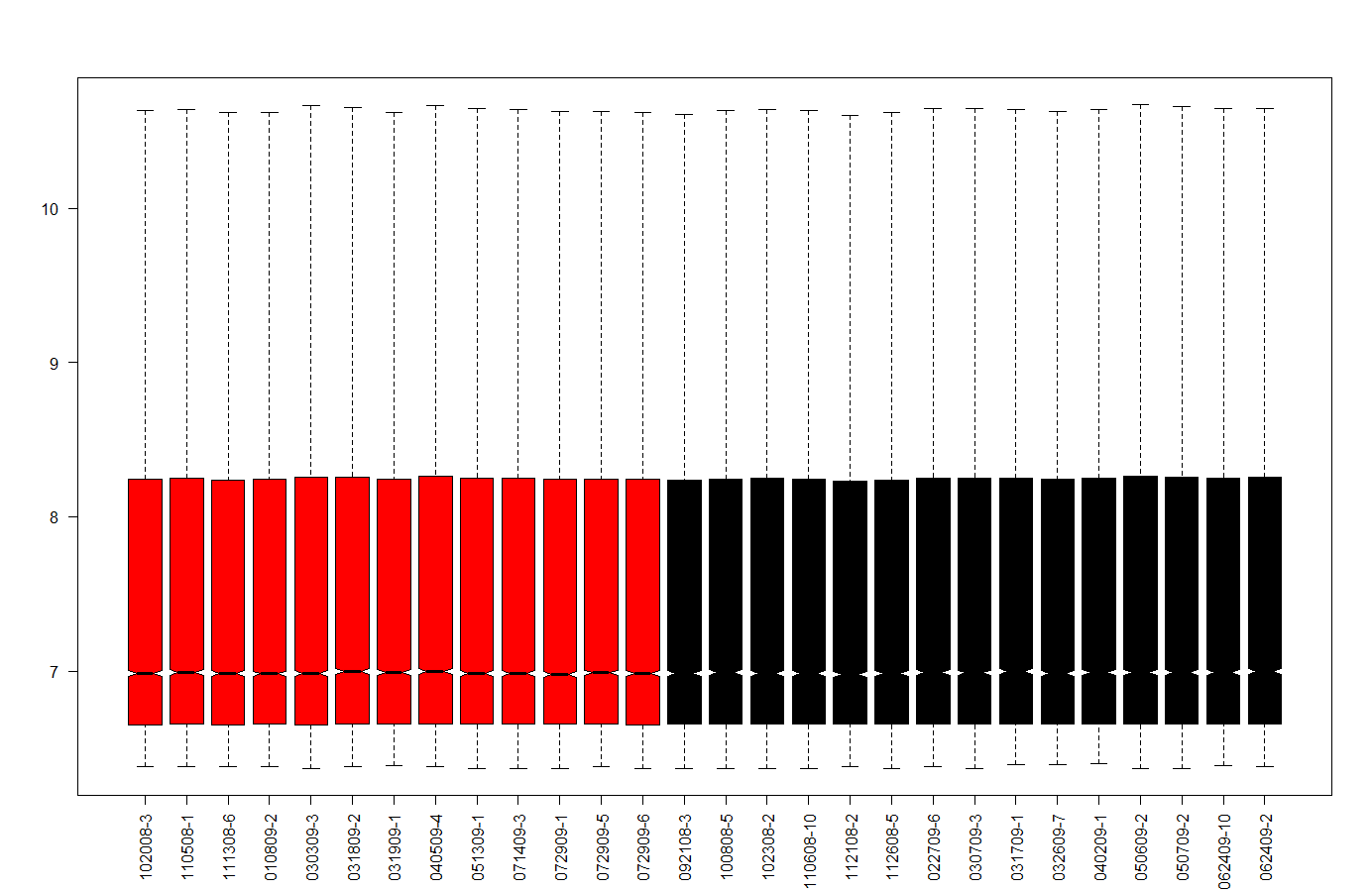

Supplement: Supplemental Information 1 — The red represents DOR samples and the black represents NOR samples. The lateral axis is the name of samples and longitudinal axis is the level of genes expression. DOR, diminished ovarian reserve; NOR, normal ovarian reserve. [file peerj-08-9812-s001.png]

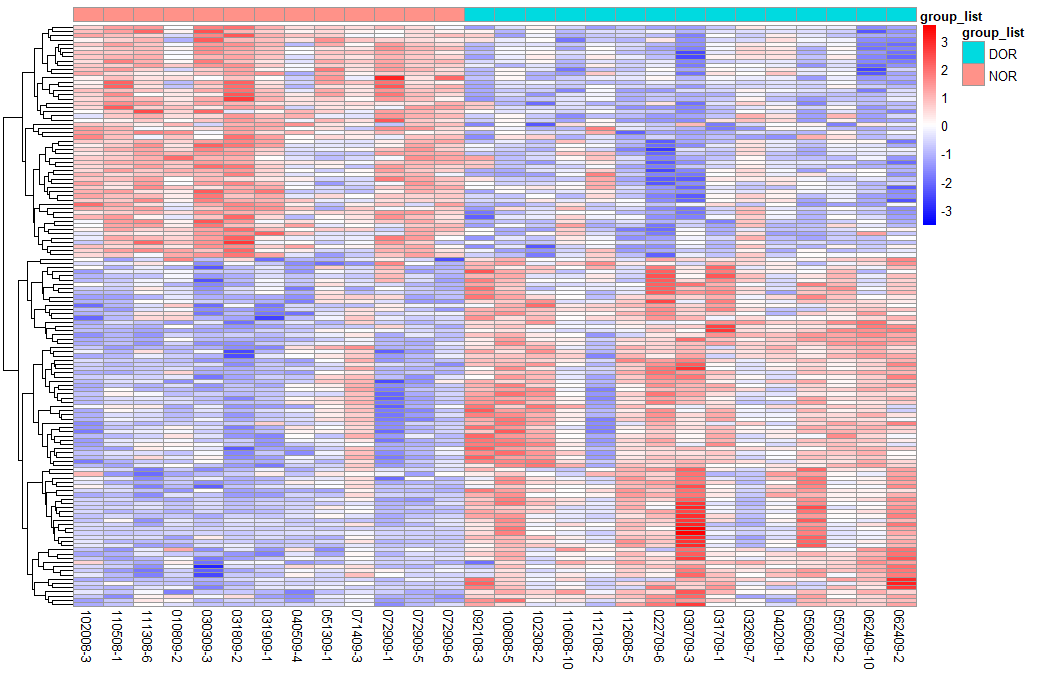

Supplement: Supplemental Information 2 — The color change from red to blue represents the values change of gene expression from high to low. DEGs, differentially expressed genes. [file peerj-08-9812-s002.png]

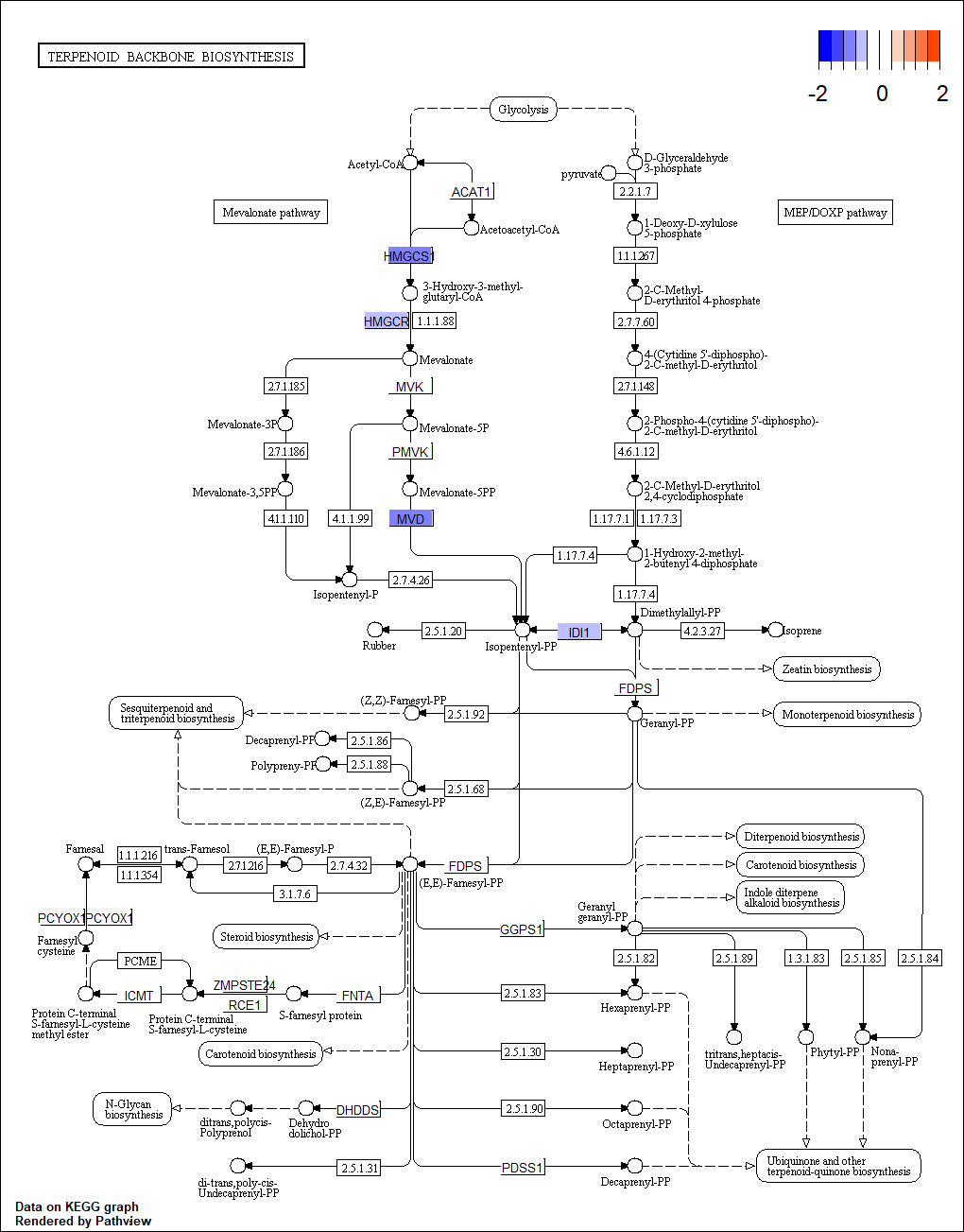

Supplement: Supplemental Information 3 — The genes with blue are downregulated DEGs. KEGG, Kyoto Encyclopedia of Genes and Genomes; DEGs, differentially expressed genes. [file peerj-08-9812-s003.png]
