# Supplementary material for: Using bioinformatics and metabolomics to identify altered granulosa cells in patients with diminished ovarian reserve
Source: PeerJ. 2020 Aug 28;8:e9812. doi: 10.7717/peerj.9812 (PMC7457930; doi:10.7717/peerj.9812)
Supplement: Supplemental Information 4 — *In publication of Woo et al., women who met two or more of the four criteria in the table except for age were defined as diminished ovarian reserve, and the parameter that was set to 7 represented for antral follicle count (AFC). [file peerj-08-9812-s004.docx]

| Age(years) | FSH (IU/L) | AMH (ng/mL) | Number of follicle on day of HCG trigger | oocytes collected of previous cycle | Cohorts |
| --- | --- | --- | --- | --- | --- |
| ≤35 | ≥8.0 | No limit | ≤7 | No limit | Skiadas et al. |
| ≤37 | ＞10 | <2 | ＜7 | ＜6 | Woo et al. * |
| ≤35 | 12≤FSH＜25 | No limit | ≤7 | No limit | Our inclusion criteria |
